# MRI Visualization of Whole Brain Macro- and Microvascular Remodeling in a Rat Model of Ischemic Stroke: A Pilot Study

**DOI:** 10.1038/s41598-020-61656-1

**Published:** 2020-03-19

**Authors:** MungSoo Kang, SeokHa Jin, DongKyu Lee, HyungJoon Cho

**Affiliations:** Department of Biomedical Engineering, Ulsan National Institute of Science and Technology, Ulsan, South Korea

**Keywords:** Neuroscience, Diseases, Medical research

## Abstract

Using superparamagnetic iron oxide nanoparticles (SPION) as a single contrast agent, we investigated dual contrast cerebrovascular magnetic resonance imaging (MRI) for simultaneously monitoring macro- and microvasculature and their association with ischemic edema status (via apparent diffusion coefficient [ADC]) in transient middle cerebral artery occlusion (tMCAO) rat models. High-resolution T_1_-contrast based ultra-short echo time MR angiography (UTE-MRA) visualized size remodeling of pial arteries and veins whose mutual association with cortical ischemic edema status is rarely reported. ΔR_2_–ΔR_2_^*^-MRI-derived vessel size index (VSI) and density indices (Q and MVD) mapped morphological changes of microvessels occurring in subcortical ischemic edema lesions. In cortical ischemic edema lesions, significantly dilated pial veins (p = 0.0051) and thinned pial arteries (p = 0.0096) of ipsilateral brains compared to those of contralateral brains were observed from UTE-MRAs. In subcortical regions, ischemic edema lesions had a significantly decreased Q and MVD values (p < 0.001), as well as increased VSI values (p < 0.001) than normal subcortical tissues in contralateral brains. This pilot study suggests that MR-based morphological vessel changes, including but not limited to venous blood vessels, are directly related to corresponding tissue edema status in ischemic stroke rat models.

## Introduction

Obstruction of the blood supply to part of the brain causes an ischemic stroke. Although diminished blood flow can result in cell death, it typically promotes two-phase cerebrovascular remodeling involving arteriogenesis and angiogenesis. Arteriogenesis, induced by physical forces such as fluid shear stress and circumferential wall stress, is a process that improves collateral circulation by vasodilating pre-existing circulatory anastomoses^1^. Vasodilation lasts until the physical forces are normalized^2^. Then, the dilated vessels are known to return to their normal diameter^3^. However, the remodeling of venous vessel size and its association with ischemic edema status is rarely investigated in the post-ischemic brain. Angiogenesis is triggered by hypoxia and induces pro-angiogenic factors that result in the sprouting of new capillaries from pre-existing vessels for maintenance or restoration of local oxygen and nutrition supplies^1,4^. In general, arteriogenesis is involved in both macro- and microvascular remodeling, while angiogenesis is a mechanism of microvascular remodeling. Adequate collateral circulation status through arteriogenesis and increased microvessel density through angiogenesis have been shown to correlate with better clinical outcomes and recovery after ischemic stroke^5–13^. However, noninvasive methods for simultaneously assessing morphological macro- and microvascular remodeling after ischemic stroke have not been established, and there is little experimental evidence of the need for such evaluation.

As a noninvasive imaging modality, magnetic resonance imaging (MRI) can provide morphological information about the vascular system. With those advantages, MRI is being widely used to investigate vascular remodeling after ischemic stroke. Time of flight MR angiography (TOF-MRA) has revealed an association between arteriogenic collateral circulation and clinical outcomes^14–16^. In a preclinical study of transient middle cerebral artery occlusion (tMCAO) in a rat model, TOF-MRA showed that macrovascular remodeling occurred at the rat brain surface region^17^. However, previous TOF-MRA studies on the ischemic stroke brains have focused mainly on arterial blood vessels, and vascular remodeling of venous pial vessels has hardly been studied to our knowledge. Also, the low spatial resolution of MRA limits the direct local morphological visualization of microvascular remodeling^1^.

On the other hand, the spatial changes of MR signals can be measured with the administration of a blood-pool contrast agent, enabling morphological mapping of the macro- and microvasculature. To obtain morphological information regarding the entire macrovasculature, high-resolution 3-dimensional T_1_-contrast-based ultra-short echo time (UTE) MRA can be used^18,19^. To obtain morphological information about the microvasculature, vessel size index (VSI) and microvessel density indices (Q and MVD) can be measured. VSI, Q, and MVD values obtained through alteration of transverse relaxation rates (ΔR_2_ and ΔR_2_^*^) due to the administration of contrast agent^20–23^ can be used to map morphological microvascular alterations after ischemic stroke^24–30^. As arteriogenesis and angiogenesis are likely to correlate with recovery after ischemic stroke, the association between vessel morphology and corresponding tissue ischemic edema status (via apparent diffusion coefficient [ADC]) can be verified by simultaneous assessment of total macro- and microvascular remodeling in the ischemic brain.

In this pilot study, we performed dual contrast MRI using superparamagnetic iron oxide nanoparticles (SPION) as a single contrast agent^18,19^. We used Monte Carlo simulation to optimize the dose of SPION by correlating ΔR_2_ and ΔR_2_^*^ values at various vessel sizes/volumes at 7-T. High-resolution (59 μm^3^) 3-dimensional UTE-MRA was combined with ΔR_2_-ΔR_2_^*^-MRI derived VSI/Q/MVD maps on tMCAO rat models. Such dual contrast MRI acquisition enabled simultaneous morphological visualization of macrovascular size remodeling at the brain surface region and microvascular size/density remodeling at the deep inner brain region. ADC values, macrovascular diameters (UTE-MRA) and microvascular size/density (VSI/Q/MVD) in cortical and subcortical regions were respectively evaluated and compared to elucidate the morphological responses of the cerebral vascular system in rat brain during recovery of ischemic stroke.

## Materials and methods

### Animal preparation

All animal experiments were approved by the Institutional Animal Care and Use Committee (IACUC) of the Ulsan National Institute of Science and Technology (UNIST) and carried out in accordance with the Animal Protection Act of Korea and Guide for the Care and Use of Laboratory Animals of the National Institutes of Health. Adult male Wistar rats (body weight 280–320 g) were housed in cages under a 12 h light/12 h dark cycle with *ad libitum* access to food and water. Rats were initially anesthetized by inhalation of 3% isoflurane in a mixture of 30% oxygen and 70% nitrous oxide and isoflurane was maintained in the range of 1~1.5% during surgery and MRI acquisition. As isoflurane and nitrous oxide is known to involve some degree of vessel dilations, vasculatures in contralateral brains were used as a reference to monitor any ipsilateral vascular alterations. Body temperature was kept constant (37 ± 1 °C) by using a warm-water circuit integrated into the animal bed and a feedback-controlled heating pad for MRI acquisition and surgery, respectively. Focal brain ischemia was induced by transient occlusion of the middle cerebral artery (MCA) according to a previously described method^31,32^ with an intraluminal monofilament (0.37 mm diameter filament, Doccol Corporation, Redlands, CA, USA). After 60 min of occlusion, the filament was withdrawn and the wound was sutured under local anesthesia. MRI acquisitions were performed on the tMCAO rats up to reperfusion day 7 after surgery.

### Microvessel size and density simulation

Monte Carlo simulation was performed to optimize the dose of SPION and examine the feasibility of vessel size-related VSI and microvessel density-related Q and MVD by comparing them with input vascular size and vascular density in experimental MRI parameters, similar to the methods used in a previous study^21^. The simulation was implemented via in-house MATLAB (MathWorks, Natick, MA) scripts, which are based on the finite perturber method (FPM)^33^ and analogous to previously described procedures^19,34,35^. Randomly oriented cylinders were generated in a 3-dimensional binary matrix (400 × 400 × 400) to represent microvessels in a tissue. The magnetic field shift (ΔB) was calculated as the sum of magnetic field shifts from all the perturbers. As simulation parameters, the main magnetic field (B_0_) was set to 7-T and the magnetic susceptibility difference between the intravascular contrast agent and nearby tissue (Δχ) was set to 4.5 × 10^−7^ (CGS unit) corresponding to a SPION dose of 360 μmol Fe/Kg (20.106 mg/Kg)^19,36^. The Monte Carlo simulation was performed consecutively for the estimation of ΔR_2_ and ΔR_2_^*^ values. Initially, 64,000,000 (400 × 400 × 400) protons were positioned uniformly within the diffusion space. In the periodic boundary condition, each proton was diffused to an adjacent position in the simulation unit time (Δt) of 1 ms, with the diffusion length of $\sqrt{2{\rm{D}}\varDelta {\rm{t}}}$ (D: diffusion coefficient, 800 μm^2^/s). The phase accumulation of each proton during diffusion time induced from different magnetic field shifts according to the position of each proton was calculated. Subsequently, ΔR_2_ and ΔR_2_^*^ values from the MR signal estimated by averaging the accumulated phase of total diffusing protons were calculated with ΔR_2_, ΔR_2_^*^ = $-\frac{\mathrm{ln}\,[{\rm{S}}({\rm{TE}})]}{{\rm{TE}}}$. TEs for the simulation and calculation of ΔR_2_ and ΔR_2_^*^ values were 8 ms and 3 ms, respectively. Based on simulated ΔR_2_ and ΔR_2_^*^ values, VSI/Q/MVD values were calculated with the following equations^20–23^:

1 $${\rm{V}}{\rm{S}}{\rm{I}}\,(\mu {\rm{m}})=0.424{({\rm{D}}/(\gamma \Delta \chi {{\rm{B}}}_{0}))}^{1/2}{({{\Delta {\rm{R}}}_{2}}^{\ast }/{\Delta {\rm{R}}}_{2})}^{3/2}$$

2 $${\rm{Q}}({{\rm{s}}}^{-1/3})={\Delta {\rm{R}}}_{2}/{({{\Delta {\rm{R}}}_{2}}^{\ast })}^{2/3}$$

3 $${\rm{MVD}}({{\rm{mm}}}^{-2})\approx {{\rm{Q}}}^{3}/(4.725{\rm{D}})$$

To simulate various microvascular conditions, different vascular radii (2–10 μm) were generated with several blood volume fractions (Bvfs) of 2%, 4%, and 6%. The MR signal simulation for each condition was repeated ten times and reported as the mean value ± standard deviation (SD).

### MRI acquisition

All MRI acquisitions were carried out on a 7-T MR scanner (Bruker, Ettlingen, Germany) with a 40-mm volume coil. Before administration of SPION, ADC, R_2_, and R_2_^*^ maps were acquired. After administration of SPION, subsequent R_2_ and R_2_^*^ maps were sequentially acquired to generate ΔR_2_ and ΔR_2_^*^ maps. Lastly, UTE-MRA was acquired after the measurement of ΔR_2_ and ΔR_2_^*^. SPION was synthesized in house^18^. The core size distribution of the iron oxide was 5 to 10 nm. Based on differential light scattering (DLS) experiment, the mean hydrodynamic diameter of the iron oxide nanoparticles was 20 ± 7 nm. The r_1_ and r_2_ of SPION were 2.36 mM^−1^s^−1^ and 32.94 mM^−1^s^−1^ at 7-T. SPION was administered as an intravenous bolus with a dose of 360 μmol Fe/Kg (optimized dose from simulation). For the experimental validations, two normal rats were scanned before and after SPION administration for UTE-MRA and two tMCAO rats were studied at 1 day after reperfusion for ΔR_2_-ΔR_2_^*^-MRI. Then, four tMCAO rats were studied at 1, 4, and 7 days after reperfusion for the quantification of macro- or microvascular remodeling with corresponding ADC measurements.

The ADC map was acquired using a diffusion-weighted echo planar imaging (EPI) pulse sequence with the following parameters: TR/TE = 3500/25.2 ms; number of averages (NA) = 8; number of segments = 3; b-values = 100, 200, 400, 600, 800, and 1000 s·mm^−2^; flip angle (FA) = 90°; matrix size = 100 × 100; field of view (FOV) = 30 × 30 mm^2^; resolution = 300 × 300 μm^2^; number of slices = 8; slice thickness = 1 mm.

The ΔR_2_ map was acquired using a multi-slice multi-echo (MSME) pulse sequence with the following parameters: TR = 6000 ms; TE = 8–160 ms; echo spacing = 8 ms; NA = 1; FA = 90°; matrix size = 256 × 256; FOV = 30 × 30 mm^2^; resolution = 117 × 117 μm^2^; number of slices = 8; slice thickness = 1 mm.

The ΔR_2_^*^ map was acquired using a multi-echo gradient echo (MEGE) pulse sequence with the following parameters: TR = 6000 ms; TE = 3–59 ms; echo spacing = 4 ms; NA = 1; FA = 90°; matrix size = 256 × 256; FOV = 30 × 30 mm^2^; resolution = 117 × 117 μm^2^; number of slices = 8; slice thickness = 1 mm.

The UTE-MRA was acquired using an UTE pulse sequence with the following parameters: TR/TE = 22/0.012 ms; NA = 1; FA = 40°; under-sampling factor = 1.13; matrix size = 512 × 512 × 512; FOV = 30 × 30 × 30 mm^3^; resolution = 59 × 59 × 59 μm^3^.

### Data processing and analysis

Data processing and analysis were performed with MATLAB, RStudio (RStudio, Boston, MA), and ImageJ (US National Institutes of Health, Bethesda, MD) software. Voxel-wise ADC values were obtained by fitting the mono-exponential decay equation S = S_0_ × exp(-ADC × b-value) with a nonlinear least-squares-fitting method.

The ΔR_2_ and ΔR_2_^*^ values were calculated by subtracting the transverse relaxation rates (R_2_ and R_2_^*^) values acquired before administration of SPION from the R_2_ and R_2_^*^ values acquired after administration of SPION. Voxel-wise R_2_ and R_2_^*^ values were obtained by fitting the mono-exponential decay equation S = S_0_ × exp(-TE × [R_2_ or R_2_^*^]). ΔR_2_ and ΔR_2_^*^ maps were used to determine VSI, Q, and MVD maps following equations (1), (2), and (3).

Acquired UTE-MRAs were denoised by application of a BM4D filter^37^. Improved signal-to-noise ratio (SNR) in BM4D filter applied UTE-MRA is shown in Supplementary Fig. S1. The brain region of each UTE-MRA was segmented using a rat brain atlas^38^ for visualization of brain vasculature only. Volume rendered UTE-MRAs were thresholded and visualized by 3DSlicer software (www.slicer.org) for the investigation of macrovascular remodeling in tMCAO rat models. The boundary value between noise and signal was set as a threshold value to delineate vasculature. In the accompanying figures, UTE-MRAs with the bluish and the yellowish regions represent brain tissue and brain vasculature, respectively. For the acquisition of vascular diameters from UTE-MRA, vasculatures in the UTE-MRA were segmented and thresholded. The same threshold value was set for each tMCAO rat model to reduce bias from diameter calculation. Based on binary images, the vascular diameters were calculated by fitting maximal spheres to every point in the vascular structure^39^ via BoneJ software^40^. The average diameter of the fitted sphere in the main vascular branch was determined as the corresponding vessel diameter. Also, the standard deviation was calculated to represent the error bar.

Cortical and subcortical regions of the ipsilateral and contralateral hemispheres were selected and segmented from the ADC map (4 or 5 slices to cover whole brain ischemic edema) of each experiment as shown in Supplementary Fig. S2. The values of ADC, VSI, Q, and MVD in the corresponding area were calculated and evaluated. Subsequently, relationship between the ADC value of the cerebral cortex and the pial venous and arterial vessel diameters (derived from UTE-MRA) were shown using a bar graph for ischemic edema (ADC_mean_ < 650 μm^2^/s) and normal tissue (ADC_mean_ > 650 μm^2^/s), respectively. Correspondingly, correlation between the subcortical ADC and VSI/Q/MVD values (derived from ΔR_2_-ΔR_2_^*^-MRI) were shown using a double box plot for ischemic edema and normal tissue, respectively. Conclusively with combined data from all experiments, Student’s t-tests were performed to assess the significant differences of MR-derived vascular morphological parameters between the ischemic edema and normal tissue. As a validation study of MR-derived VSI, 3-dimensional mouse brain microvascular data from the knife-edge scanning microscope (KESM) brain atlas (http://kesm.cs.tamu.edu/home/index.php) was used^41,42^. The vasculature was selected by thresholding. Based on binary images, microvascular diameters were calculated by BoneJ software.

## Results

### High-resolution UTE-MRA

**Figure 1:**
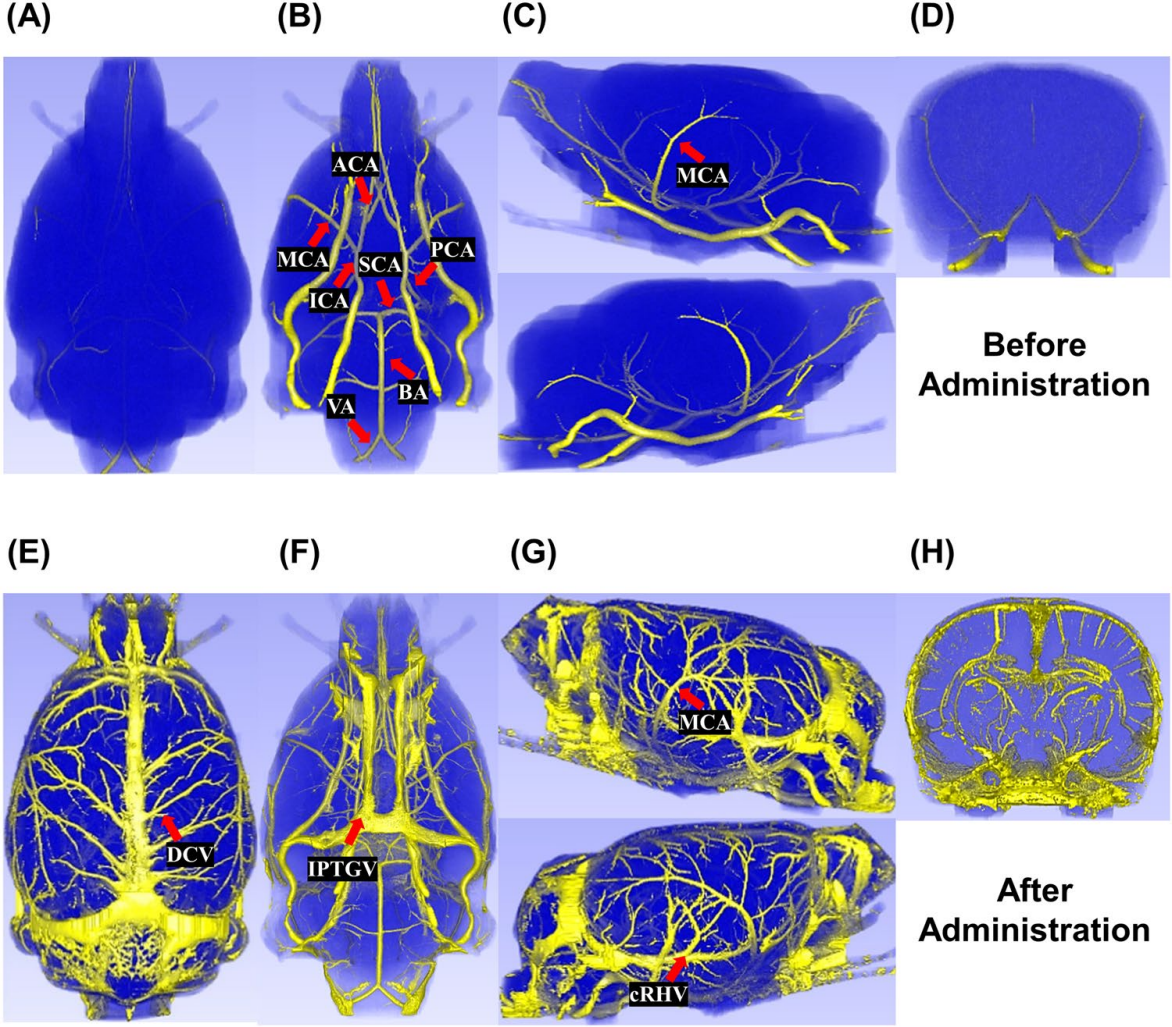
UTE-MRAs of two normal rat brain. Dorsal views (**A** and **E**), ventral views (**B** and **F**), lateral views (**C** and **G**) and anterior-to-posterior views (**D** and **H**) of UTE-MRAs acquired before and after administration of SPION, respectively. Red arrows indicate middle cerebral artery (MCA), posterior cerebral artery (PCA), internal carotid artery (ICA), anterior cerebral artery (ACA), superior cerebellar artery (SCA), vertebral artery (VA), basilar artery (BA), dorsal cerebral vein (DCV), interpterygoid emissary vein (IPTGV), and caudal rhinal vein (cRHV). Figure was generated from 3DSlicer (4.5.0–1, www.slicer.org).

Figure 1 shows two normal volume-rendered rat brains acquired from high-resolution UTE-MRAs. Before administration of SPION, no cerebral veins are visible in the dorsal view (Fig. 1A). In contrast, major arteries composing and contributing to the circle of Willis are visible in the ventral view (Fig. 1B, red arrows). In lateral views (Fig. 1C, red arrow), ascending MCAs are visible. No vessels are visible in the anterior-to-posterior view (Fig. 1D). UTE-MRA before administration of SPION is a TOF-MRA, which shows large arteries populating the brain surface region. After administration of SPION, not only major arteries but also veins including dorsal cerebral vein (DCV), interpterygoid emissary vein (IPTGV), and caudal rhinal vein (cRHV) are visible in the dorsal (Fig. 1E, red arrow), ventral (Fig. 1F, red arrow), and lateral views (Fig. 1G, red arrows). In the anterior-to-posterior view (Fig. 1H), intracortical penetrating and other macro-vessels are shown.

**Figure 2:**
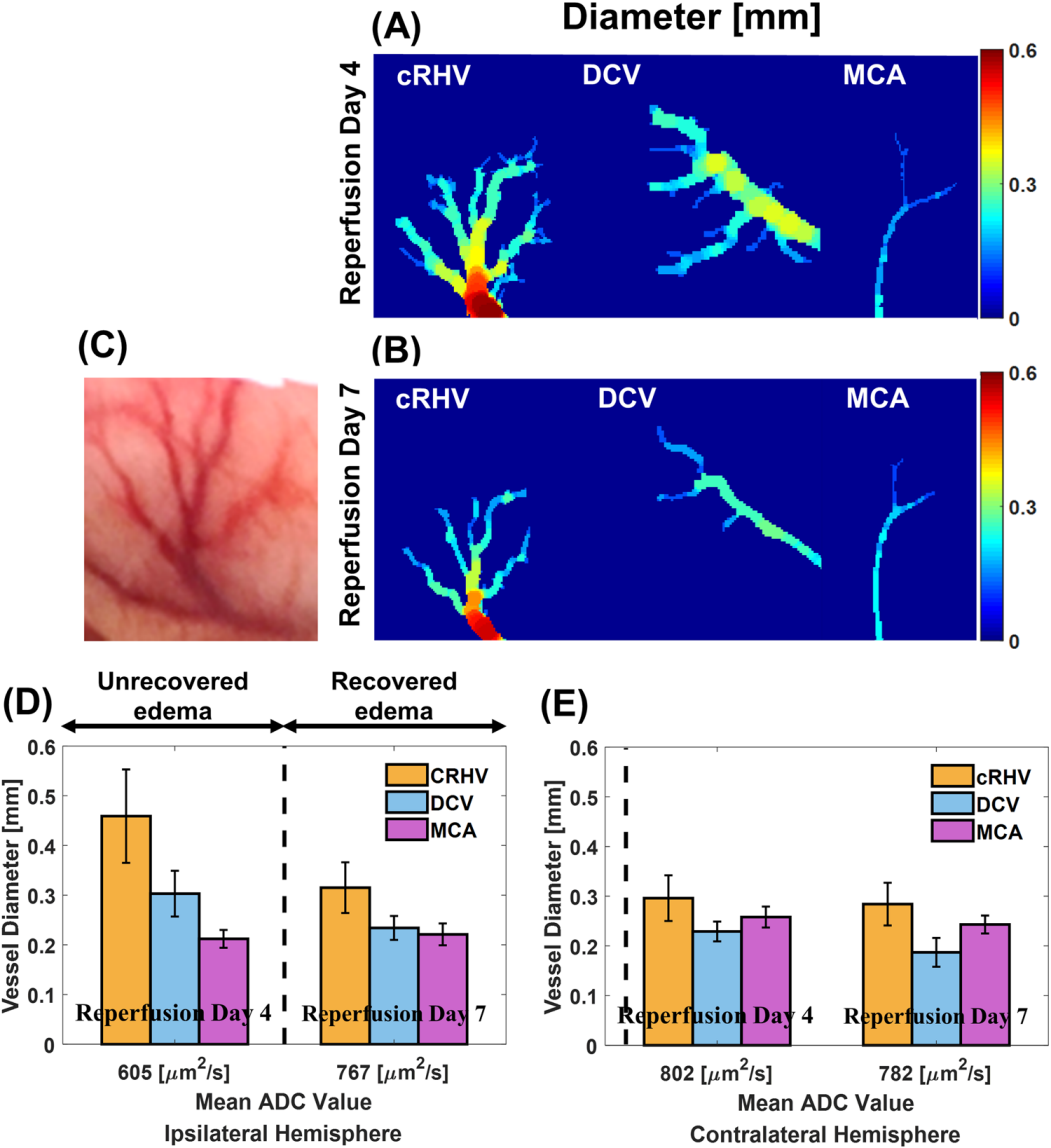
Macrovascular remodeling of tMCAO rat brain. The results of diameter fittings of cRHV, DCV and MCA in ipsilateral hemisphere at reperfusion day 4 (**A**) and at reperfusion day 7 (**B**). Corresponding snapshot of cRHV at reperfusion day 7 (**C**). The mean value of fitted sphere diameters of the main branch was set as respective vessel diameter. Vessel diameters of cRHV (orange), DCV (blue), and MCA (purple) of the ipsilateral hemisphere with respect to mean ADC values of the corresponding cortical lesion at reperfusion days 4 and 7 (**D**). Vessel diameters of cRHV (orange), DCV (blue), and MCA (purple) of the contralateral hemisphere with respect to mean ADC values of the corresponding cortical region at reperfusion days 4 and 7 (**E**). The dotted vertical lines mark the ADC = 650 μm^2^/s, which are set to separate ischemic edema from recovered/normal tissue. Figures (**A,B**) were analyzed using BoneJ (www.bonej.org) within ImageJ (1.51 g, imagej.nih.gov/ij/) and generated from MATLAB (R2017b, www.mathworks.com).

To directly visualize alterations of macrovasculature in ipsilateral hemisphere of UTE-MRAs, vessel diameter maps of cRHV, DCV and MCA in the ipsilateral hemisphere at reperfusion day 4 and day 7 are shown in Fig. 2A,B. As the illustrations for morphological changes of macrovasculature in the tMCAO model, longitudinally acquired UTE-MRAs and ADC maps of re-perfused tMCAO rat brains are fully shown in Supplementary Fig. S3. Bar graphs show mean ADC values of cortical regions along with the diameters of cRHV, DCV, and MCA in the ipsilateral and contralateral hemispheres at reperfusion day 4 (ischemic edema in the ipsilateral brain) and at reperfusion day 7 (recovered ischemic edema in the ipsilateral brain) as shown in Fig. 2D,E, respectively. Only the main branches of vasculatures were used to calculate mean vessel diameter to minimize measurement errors. A significant dilation of the pial venous (DCV and cRHV) vessels in the ipsilateral region is evident at reperfusion day 4 and reduced to normal diameters at reperfusion day 7, followed by recovery of the corresponding mean ADC value as shown in Fig. 2D. A snapshot of the corresponding cRHV is shown in Fig. 2C at reperfusion day 7 as a reference for direct comparison with UTE-MRA. No significant variations were observed for both cRHV and DCV in the contralateral brain (Fig. 2E). As shown in Fig. 2D,E, the pial arterial (MCA) vessel thinned at reperfusion day 4 was restored to its normal diameter at reperfusion day 7 when compared to that of the contralateral brain.

### ΔR_2_-ΔR_2_^*^-MRI

**Figure 3:**
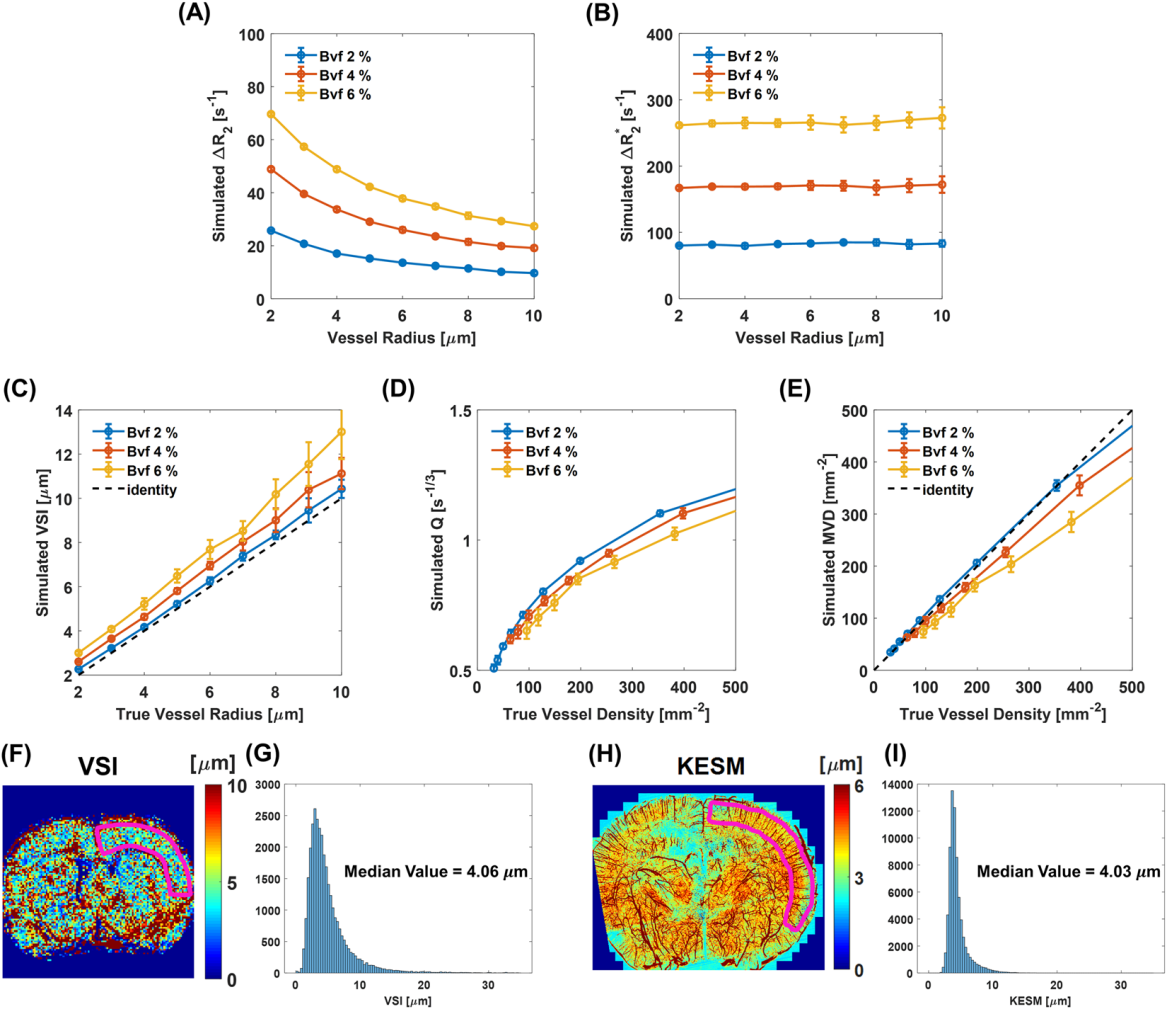
Monte Carlo simulation results. Vessel radius and blood volume fraction (Bvf) dependent simulated ΔR_2_ (**A**) and ΔR_2_^*^(**B**). Comparison of simulated VSI with true vessel radius (**C**). Comparisons of simulated Q and MVD with true vessel density (**D**,**E**, respectively). Experimental VSI map (**F**) and distribution (**G**) in the cortical region (ROI, marked with purple line in **F**). Corresponding gold-stand vessel size map (**H**) and distribution (**I**) in cortical region from knife-edge scanning microscope (KESM). Figure (**F**) was generated from MATLAB (R2017b, www.mathworks.com). Figure (**H**) was analyzed using BoneJ (www.bonej.org) within ImageJ (1.51 g, imagej.nih.gov/ij/) and generated from MATLAB (R2017b, www.mathworks.com).

Vessel radius and Bvf-dependent ΔR_2_ and ΔR_2_^*^ values from the Monte Carlo simulation are shown at 7-T with experimental SPION dose and MRI parameters in Fig. 3A,B, respectively. As vessel radius increased, simulated ΔR_2_ values decreased while simulated ΔR_2_^*^ values were approximately constant in all Bvf conditions. Also, as Bvf increased, simulated ΔR_2_ and ΔR_2_^*^ values increased in the same vessel radius condition. Simulated VSI (Fig. 3C) showed insignificant dependency on Bvf and increased as a function of the input vessel radius. Simulated Q and MVD values increased as a function of input vessel density, irrespective of Bvf (Fig. 3D,E). Experimental VSI values distribution in the cortical region (ROI is shown in Fig. 3F) is shown in Fig. 3G. In direct comparison, the gold-standard vessel size distribution of the cortical region (ROI is shown in Fig. 3H) from KESM is shown in Fig. 3I and shows a consistent long-tail toward larger values distribution with respect to the experimental VSI values distribution. Median values of VSI and KESM were 4.06 μm and 4.03 μm, respectively. Although fitted echo trains showed low ΔR_2_ values^21^, those values showed less fluctuation compared to ΔR_2_ values from the first echo (Supplementary Fig. S4). ΔR_2_ values (~20 s^−1^) from simulated (Bvf 2%, radii 2–4 μm) and the first echo of MSME acquisition were consistent as shown in Supplementary Fig. S4. For VSI/Q/MVD calculation, robust ΔR_2_ maps obtained from fitted echo trains were multiplied by factor of 4.375 (determined from experiments) and used for VSI/Q/MVD input to be consistent with simulations.

**Figure 4:**
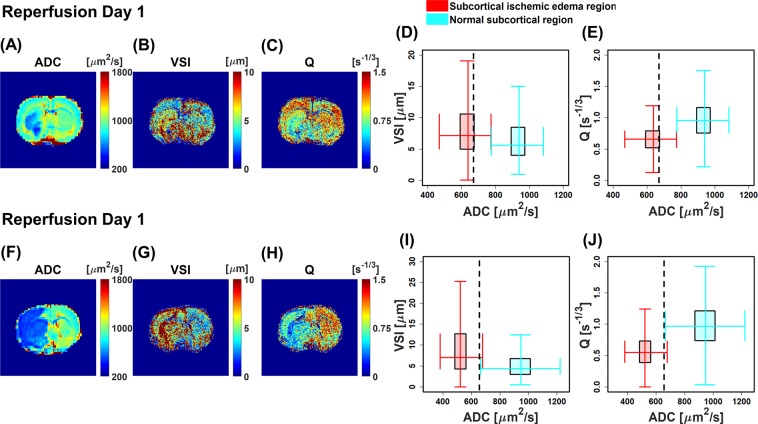
Microvascular remodeling of two tMCAO rat brains. ADC maps (**A** and **F**), VSI maps (**B** and **G**), and Q maps (**C** and **H**) of two tMCAO rat brains at reperfusion day 1. Double box plots between VSI values and corresponding ADC values of subcortex for both rats (**D** and **I**). Double box plots between Q values and corresponding ADC values of subcortex for both rats (**E** and **J**). The dotted vertical lines mark the ADC = 650 μm^2^/s, which are set to separate ischemic edema from normal tissue. Figures (**A–C**) and (**F–H**) were generated from MATLAB (R2017b, www.mathworks.com).

As the illustrations for morphological changes of microvasculature in the tMCAO model, ADC, VSI, and Q maps of two tMCAO rat brains acquired at early reperfusion day (day 1) are shown in Fig. 4. The two tMCAO rat brains show different ischemic edema size in the ipsilateral hemisphere of the ADC maps (Fig. 4A,F). In each ipsilateral ischemic edema lesion, the corresponding VSI map showed increased VSI values than the corresponding region in the contralateral hemisphere (Fig. 4B,G). In contrast, each Q map showed lower values compared to those of the corresponding region in the contralateral hemisphere (Fig. 4C,H). The location of abnormal ADC, VSI, and Q values appeared to be strongly co-localized. Double box plots (VSI versus ADC and Q versus ADC) verified increased VSI values and decreased Q values in the subcortical ischemic edema lesions (ADC_mean_ < 650 μm^2^/s) of the ipsilateral side compared with those of the contralateral side (Fig. 4D,E,I,J).

### Dual contrast MRI

**Figure 5:**
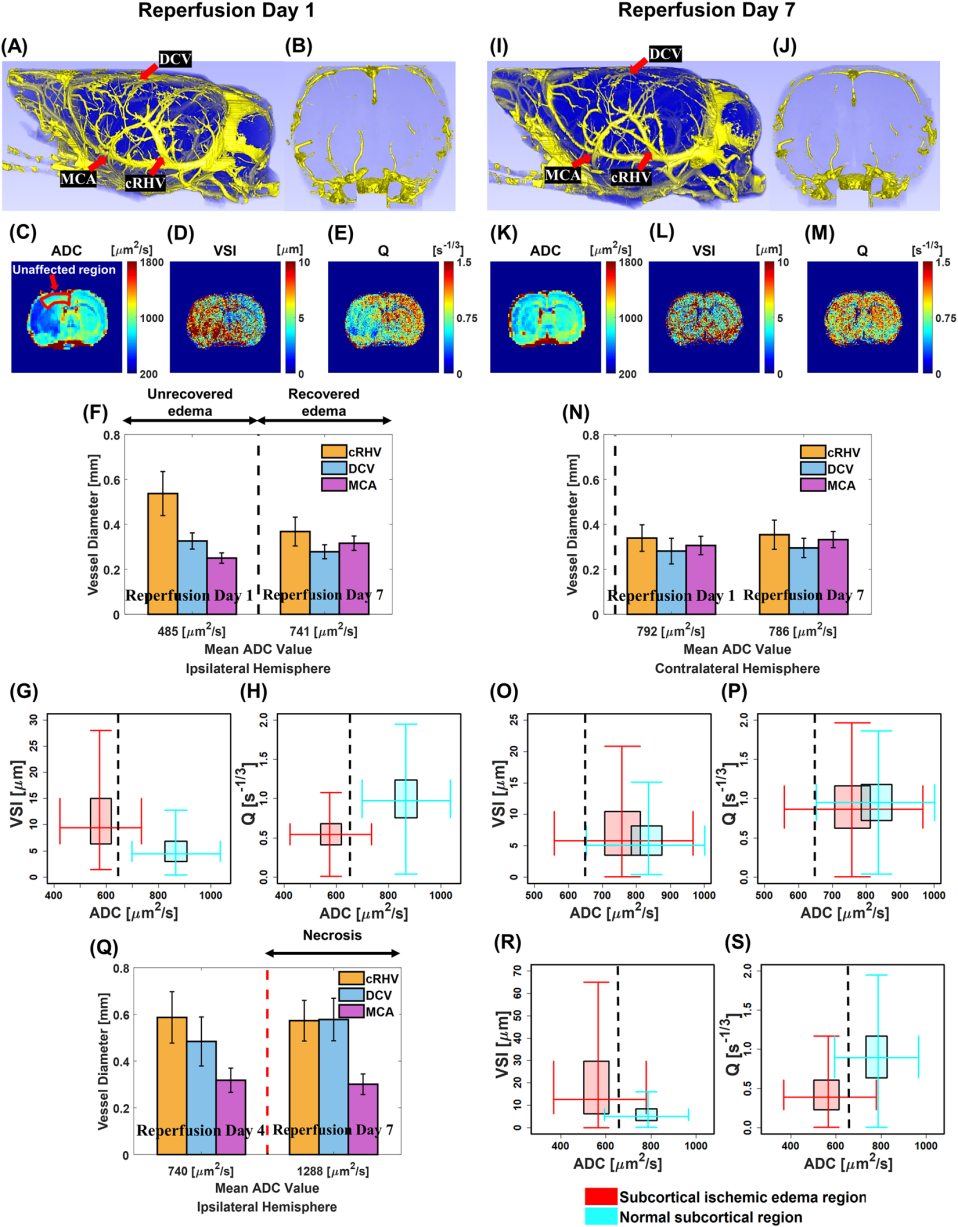
Macro- and microvascular remodeling of tMCAO rat brain. Lateral views of ipsilateral hemisphere (**A** and **I**) and anterior-to-posterior views (**B** and **J**) of tMCAO rat brain UTE-MRAs and corresponding ADC maps (**C** and **K**), VSI maps (**D** and **L**), and Q maps (**E** and **M**) of tMCAO rat brain acquired at reperfusion days 1 and 7, respectively. Vessel diameters of cRHV (orange), DCV (blue), and MCA (purple) of the ipsilateral hemisphere with respect to mean ADC values of the corresponding cortical lesion at reperfusion days 1 and 7 (**F**). Corresponding behavior of necrotic cortex in a different animal (**Q**). Vessel diameters of cRHV (orange), DCV (blue), and MCA (purple) of the contralateral hemisphere with respect to mean ADC values of the corresponding cortical region at reperfusion days 1 and 7 (**N**). Double box plots between VSI values and corresponding ADC values of subcortex (**G** and **O**) for reperfusion days 1 and 7, respectively. Corresponding behavior for persisting edema in another different animal at reperfusion day 7 (**R**). Double box plots between Q values and corresponding ADC values of subcortex (**H** and **P**) for reperfusion days 1 and 7, respectively. Corresponding behavior for persisting edema in another different animal at reperfusion day 7 (**S**). The black dotted vertical lines mark the ADC = 650 μm^2^/s, which are set to separate ischemic edema from recovered/normal tissue. The red dotted vertical line in (**Q**) separates recovered tissue from necrotic tissue. Figures (**A**,**B**), (**I**), and (**J**) were generated from 3DSlicer (4.5.0–1, www.slicer.org). Figures (**C–E**) and (**K–M**) were generated from MATLAB (R2017b, www.mathworks.com).

Longitudinal dual contrast MRI results, which reflected macro- and microvascular remodeling in three tMCAO rat models, are shown in Fig. 5 and supplementary Figs. S5 and S7. Even though each tMCAO rat model showed heterogeneous evolution after reperfusion, distinct vascular size alterations and density reductions at early reperfusion days (day 1~day 4) were consistently observed in the area of ipsilateral ischemic edema. After such alterations, morphological normalization of the pial vessels and microvessels was observed to be associated with the recovery of respective ADC values in the cerebral cortex and subcortex at late reperfusion day (day 7).

Representatively, we show a case of normal recovery of both cortical and subcortical lesions after reperfusion and explain the association of morphological vascular normalizations with ADC values. UTE-MRAs showed normal thinning of a dilated cRHV and DCV in lateral views of the ipsilateral hemisphere as reperfusion continued from day 1 to day 7 (Fig. 5A,I, red arrows). Corresponding cortical ADC values in the ischemic edema region increased as reperfusion continued from day 1 to day 7 (Fig. 5C,K). Consistently, VSI values in the ischemic edema lesion decreased and Q values increased and returned to normal range, comparable to those of the contralateral hemisphere at reperfusion day 7 (Fig. 5D,E,L,M). Subcortical ADC values in the ischemic edema region also increased to the normal range as reperfusion continued from day 1 to day 7 as well (Fig. 5C,K). Figures 5B,J show brain inner regions of UTE-MRAs as the same slice position and thickness with VSI/Q maps. Abnormality of vessels in subcortical ischemic edema region is more distinctly visible in VSI/Q maps compared to brain inner region of UTE-MRA at reperfusion day 1 (Fig. 5B,D,E).

In the cortical area, bar graphs show mean ADC values along with the diameters of cRHV, DCV, and MCA located in the ipsilateral (Fig. 5F) and contralateral (Fig. 5N) hemisphere at day 1 (ischemic edema (ADC_mean_ < 650 μm^2^/s) in cortex and subcortex) and day 7 (recovered ischemic edema in ipsilateral brain) after reperfusion, respectively. A significant dilation of cRHV in the ipsilateral area was evident at reperfusion day 1, decreased to the normal diameter at reperfusion day 7, and corresponding ADC value at reperfusion day 7 was restored. On the other hand, DCV showed unimpressive variations, consistent with the fact that no significant ADC reduction was observed upper cortical region marked by a red arrow in Fig. 5C. The reduction of MCA diameter in the ipsilateral side at reperfusion day 1 was normalized at reperfusion day 7. On the contrary, when markedly elevated ADC values of the cortical area at reperfusion day 7 and day 13 (Supplementary Fig. S5K, white arrow and Supplementary Fig. S6) were apparent with a clear indication of necrosis for a different animal, no significant diameter change of those vessels is observable between reperfusion days 4 and 7 (Supplementary Fig. S5A and S5I, red arrows) as shown in Fig. 5Q. Thus, macrovascular normalization was observed for recovering ADC values, but persisting macrovascular alterations were apparent along with non-recovering ADC values in cortical ischemic edema region.

In the subcortical area, double box plots show increased VSI values (Fig. 5G) and reduced Q values (Fig. 5H) in ipsilateral ischemic edema lesion compared to those in the contralateral region at reperfusion day 1. However, the VSI (Fig. 5O) and Q (Fig. 5P) values in ipsilateral lesion became similar to those of the contralateral region at reperfusion day 7. Corresponding ADC values in the subcortical ischemic edema region were restored. On the contrary, when non-recovering ADC values in subcortical ischemic edema region is persistent as observed from another different animal (Supplementary Fig. S7K), double box plots in Fig. 5R,S show increased VSI and decreased Q values in ipsilateral ischemic edema lesions compared to those in the contralateral region even at 7 days after reperfusion. Thus, microvascular normalization was observed for recovering ADC values, but persisting microvascular alterations were apparent along with non-recovering ADC values in subcortical ischemic edema region. The need for dual contrast MR imaging is again emphasized in the purpose of simultaneously investigate heterogeneous macro- and microvascular remodeling in ischemic stroke associated with the progression of ischemic edema.

### Macro- and microvascular remodeling analysis

**Figure 6:**
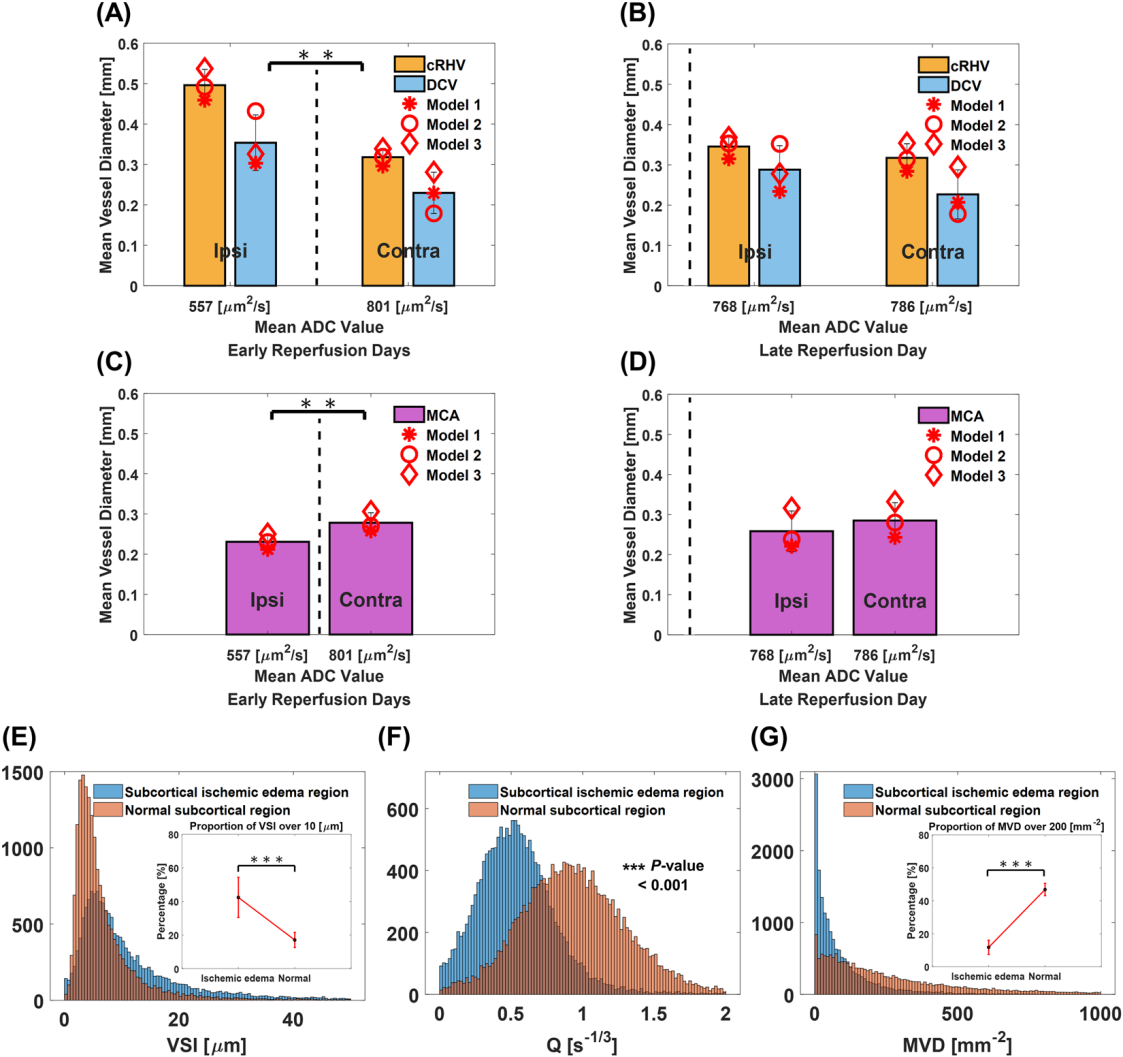
Statistical analysis of macro- and microvascular remodeling. Mean vessel diameters of combined maximum cRHVs and DCVs diameters at early days (from day 1 to day 4) of reperfusion (**A**), three tMCAO rat models marked with star, circle, and diamond symbols. Mean vessel diameters of combined cRHVs and DCVs at reperfusion day 7 (**B**), three tMCAO rat models marked with star, circle, and diamond symbols. Mean vessel diameter of combined minimum MCAs at early days (from day 1 to day 4) of reperfusion (**C**), three tMCAO rat models marked with star, circle, and diamond symbols. Mean vessel diameter of combined MCAs at reperfusion day 7 (**D**), three tMCAO rat models marked with star, circle, and diamond symbols. Maximum cRHVs and DCVs and minimum MCAs diameters were chosen between early reperfusion days. Corresponding mean ADC values of the cortex are shown. One tMCAO rat model with cortical necrosis at reperfusion day 7 (Supplementary Fig. S5) was excluded from macrovascular remodeling analysis. Histograms of combined VSI values (total 5 tMCAO rat models with six cases for ischemic edema and ten cases for recovered/normal were summarized) in ischemic edema and normal regions of subcortex (**E**). The proportions of larger VSI values (>10 μm) in ischemic edema and normal regions of subcortex are respectively shown in the inset figure of E. Histograms of combined Q and MVD values in ischemic edema and normal regions of subcortex (**F,G**, respectively). The proportions of larger MVD values (>200 mm^−2^) in ischemic edema and normal regions of subcortex are respectively shown in the inset figure of G. ***(p < 0.001) and **(p < 0.01) from Student’s t-test. The dotted vertical lines mark the ADC = 650 μm^2^/s, which are set to separate ischemic edema from recovered/normal tissue.

In the cortex, the maximum vessel diameters of cRHV and DCV and the minimum vessel diameter of MCA in the ipsilateral brain were respectively combined at early reperfusion days (days 1 and 4), except for the case of cortical necrosis progression. Consistently, the vessel diameters of normalizing cRHV, DCV, and MCA in the ipsilateral brain were respectively combined at late reperfusion day (day 7). Corresponding vessel diameters of the contralateral region were also combined, accordingly. Then, the association between mean ADC values of cortical regions and morphological alteration of pial venous and arterial vessels from UTE-MRAs were observed. Mean diameters of venous cRHVs and DCVs were significantly (p = 0.0051, paired Student’s t-test with combined cRHV and DCV diameters) dilated in the lesions of ischemic edema at early reperfusion days and then normalized at late reperfusion day with the restoration of corresponding ADC values as shown in Fig. 6A,B, respectively. On the contrary, the mean diameter of arterial MCAs was significantly (p = 0.0096, paired Student’s t-test) thinned at early reperfusion days and then recovered at late reperfusion day as shown in Fig. 6C,D, respectively.

In the subcortex, the VSI/Q/MVD values in ischemic edema lesions (ADC_mean_ <650 μm^2^/s) and those values in normal tissue of contralateral hemisphere (ADC_mean_ > 650 μm^2^/s, recovered ipsilateral area included) were also respectively combined for all cases. Then, the association between ADC values and VSI/Q/MVD values from ΔR_2_-ΔR_2_^*^-MRI was also revealed. The VSI values showed a non-Gaussian distribution with a long tail toward larger values as shown in Fig. 6E. Compared with normal tissues, VSI values in ischemic edema lesions showed wider distribution with significantly (p < 0.001, unpaired Student’s t-test) increasing relative proportion of larger vessels (VSI > 10 μm). The proportion of small vessels (VSI < 10 μm) was observed to be relatively reduced. The Q values of ischemic edema lesions were significantly (p < 0.001, unpaired Student’s t-test) smaller than those of values in the normal areas as shown in Fig. 6F. MVD values in ischemic edema lesions showed narrower distribution with significantly (p < 0.001, unpaired Student’s t-test) reduced relative proportion of larger vessel densities (MVD > 200 mm^−2^) as shown in Fig. 6G.

## Conclusions and discussion

To quantitatively evaluate macro- and microvascular remodeling after ischemic stroke, we performed dual contrast MRI on tMCAO rat models. To estimate association of vascular remodeling with ischemic edema status, ADC maps were also acquired, which quantitatively represent ischemic tissue status^43–45^. T_1_-contrast-based UTE-MRAs visualized morphological alterations of the macrovasculature occurring at the tMCAO rat brain surface region at an isotropic resolution of 59 μm^3^. In response to MCA occlusion, dilated pial venous vessels (cRHV and DCV) were clearly observed at early reperfusion days (1 to 4 days) as collateral circulatory role likely to accommodate thinned MCA, reflecting the early phase of venous macrovascular dilation. After this dilation, reduced venous vessel diameters were visible (7 days after reperfusion), which correlated with the restoration of ADC values. In contrast, thinned pial arterial vessel (MCA) was observed at early reperfusion days and restored at late reperfusion day (7 days). Because MCA was occluded initially, it is likely that the size of MCA was observed to be reduced even at early days of reperfusion and gradually recovered along with ADC recovery. Our observations on the different morphological response of pial arterial and venous vessels during the ischemic edema recovery would still require further investigations for physiological origins.

At the same time, the utilization of VSI/Q/MVD maps, which were derived from ΔR_2_-ΔR_2_^*^-MRI, verified morphological alterations of the microvasculature occurring at the tMCAO rat brain inner region. As a comparison of our VSI/Q/MVD values with other previous validation studies, cortical regions of contralateral hemispheres for all tMCAO rat models were segmented and combined. Such median values of VSI/Q/MVD from all tMCAO rat models were 4.06 μm, 1.07 s^−1/3^, and 337.4 mm^−2^, respectively. Those values are quantitatively consistent with other validation reports^21,26,46^. Consistent with macrovascular remodeling, comparison VSI/Q/MVD values with subcortical ischemic edema status revealed an increased proportion of larger VSI values in the subcortical ischemic edema lesions. Also, reduced Q and proportion of larger MVD values were observed in the subcortical ischemic edema lesions, which are qualitatively consistent with other reports^24,27,47^. Microvascular normalization was observed at late reperfusion day (day7) as well. On the other hand, simultaneous morphological characterization of macro- and microvasculature showed a significant spatial variation of vascular changes. In some cases, even when cortical macrovascular remodeling was normal, abnormal subcortical microvascular remodeling was observed to correlate with unrestored ischemic edema and vice versa. The importance of such whole-brain monitoring of the cerebral vasculature and their association with ischemic recovery in multiple length-scales is illustrated in ischemic stroke brains.

It is also important to note that morphological changes of venous vessels in the post-ischemic stroke brain were rarely reported before. Considering the fact that venous macrovascular dilations were observed from UTE-MRAs in early reperfusion days, and that the venous cerebral volume fraction reaches 70% in the rodent brain^48^, observed changes of VSI/Q/MVD values may be partially attributed to changes in venous microvasculature in subcortex area as well. Because the ischemic edema (via the ADC) is observed to be significantly associated with changes in MR-derived morphological (size and density) macro- and microvasculature, multi-length vascular information, including venous vasculature may help optimize the drug or treatment strategy after ischemic stroke. However, further quantification of sole venous microvascular information in the ischemic brain requires a technical ability to differentiate the arterial and venous systems of the microvasculature.

The difference in the distribution of VSI values between the subcortical ischemic edema lesion and the normal region is informative. In our Monte Carlo simulation, each VSI, Q, and MVD value was calculated from a single microvessel radius condition. However, in real cerebrovascular systems, various vascular radii may co-exist, which may complicate direct interpretation of MR-derived microvascular parameters. For example, in Monte Carlo simulation, reduction of relatively small vessels or actual dilation of all vessels may provide similarly increased VSI values. Also, if distributions of relatively small and large vessels are reduced in similar proportions, VSI values may not change. In this case, Q and MVD values should provide complementary information. Experimentally, a significant decrease in the Q and MVD values in ischemic edema lesions observed with changes in the VSI distribution supports the hypothesis that a number of small vessels decreases and a number of large vessels increases in the ischemic edema lesions. To establish the effectiveness of MRI-derived microvascular remodeling data for analyzing ischemic stroke progression, a validation study with microvessel size distributions may be required, using proven techniques from the previous studies^26,28,49^.

This study also suggests areas for possible technical improvements. First, as the current method enables sequential acquisition of perfusion parameters from DSC-MRI as well, combining diffusion and vascular morphological and functional information may provide a more complete picture of ischemic stroke progression, requiring further optimization of acquisition protocols. Second, high-resolution dual contrast MRI requires relatively long scan times, which hampers its routine application yet. For reduction of scan time, adjusting resolution with MR parameters that can affect scan time is necessary. There are several techniques, such as compressed sensing^50^, parallel imaging^51^, and super-resolution reconstruction via deep learning^52,53^, that can additionally reduce scan time. Further study is required to verify the effectiveness of those techniques for clinical application of dual contrast MRI.

In summary, dual contrast MRI with SPION as a single contrast agent can be successfully performed on rat models of tMCAO to simultaneously visualize whole brain macro- and microvascular remodeling after ischemic stroke, including pial venous vessels. Visualization and quantification of such simultaneous macro- and microvascular remodeling indicated that MR-based morphological (size and density) vessel normalization is directly associated with restored ADC values in post-ischemic stroke rat brains. Multiscale monitoring of the cerebrovascular system with dual contrast MRI may further elucidate the vascular mechanism of ischemic stroke recovery.

## Supplementary Information

Supplementary Information.

## Data Availability

The datasets generated and analyzed during the current study are available from the corresponding author on reasonable request.
