# Supplementary material for: MRI Visualization of Whole Brain Macro- and Microvascular Remodeling in a Rat Model of Ischemic Stroke: A Pilot Study
Source: Sci Rep. 2020 Mar 19;10:4989. doi: 10.1038/s41598-020-61656-1 (PMC7081185; doi:10.1038/s41598-020-61656-1)
Supplement: Supplementary file 1 — Supplementary Information. [file 41598_2020_61656_MOESM1_ESM.pdf]

MRI Visualization of Whole Brain Macro- and Microvascular Remodeling in a Rat Model of Ischemic Stroke: A Pilot Study

MungSoo Kang[1], SeokHa Jin[1], DongKyu Lee[1], and HyungJoon Cho[1,]*

[1] Department of Biomedical Engineering, Ulsan National Institute of Science and Technology, Ulsan, South Korea

*Corresponding author.

Name: HyungJoon Cho

Mailing address: Unist-gil 50 (100 Banyeon-ri), Eonyang-eup, Uljugun, Ulsan Metropolitan City, Republic of Korea 689-798

Phone: 82-052-217-5204, Fax: 82-052-217-2509

Email: hjcho@unist.ac.kr

Supplementary Figures

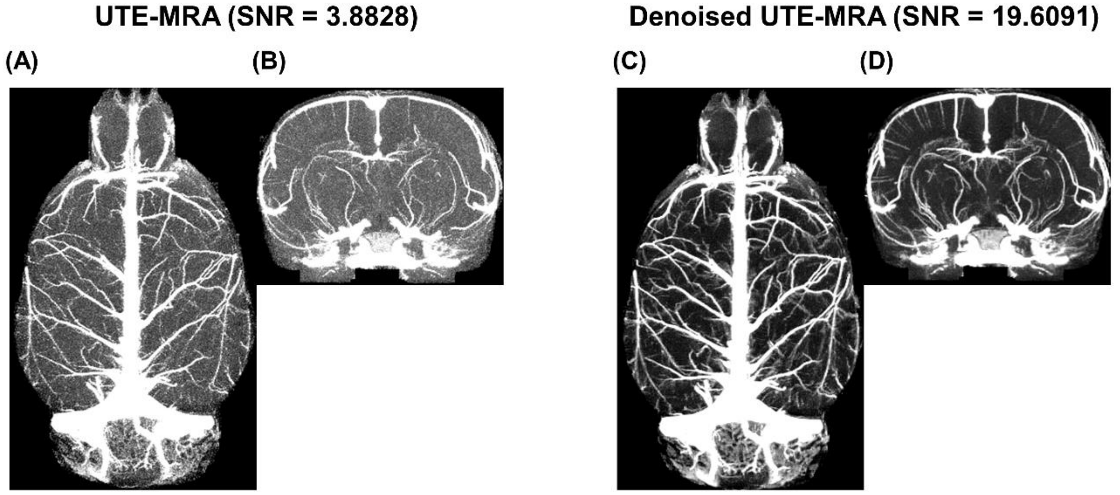

Figure S1. Original and BM4D filter applied UTE-MRAs. Dorsal views (A and C) and anterior-to-posterior views (B and D) of normal rat brain UTE-MRA and BM4D filter applied UTE-MRA with 60-slices maximum intensity projection and application of the same threshold value, respectively. SNR values of 3.8828 and 19.6091 for UTE-MRA and BM4D filter applied UTE-MRA, respectively. Figure was generated from MATLAB (R2017b, www.mathworks.com).

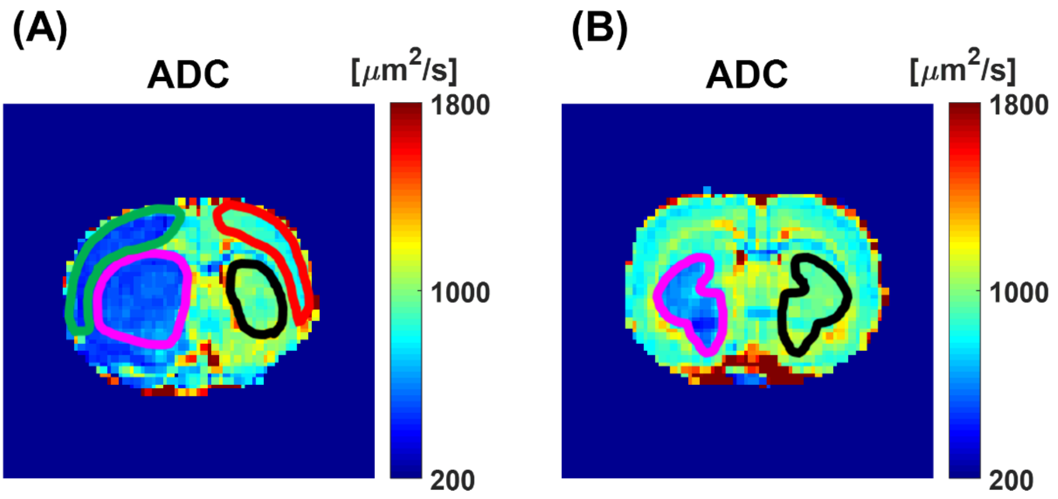

Figure S2. Scheme for cortex and subcortex segmentation. Representative ROIs for cortical and subcortical ischemic edema regions in ipsilateral hemisphere and corresponding normal regions in the contralateral hemisphere (A). Representative ROIs for subcortical ischemic edema region in ipsilateral hemisphere and corresponding normal region in contralateral hemisphere (B). A green line indicates ROI of cortical ischemic edema region in ipsilateral hemisphere, a red line indicates ROI of corresponding cortical normal region in contralateral hemisphere, purple lines indicate ROIs of subcortical ischemic edema regions in ipsilateral hemispheres, and black lines indicate ROIs of corresponding subcortical normal regions in contralateral hemisphere. Figure was generated from MATLAB (R2017b, www.mathworks.com).

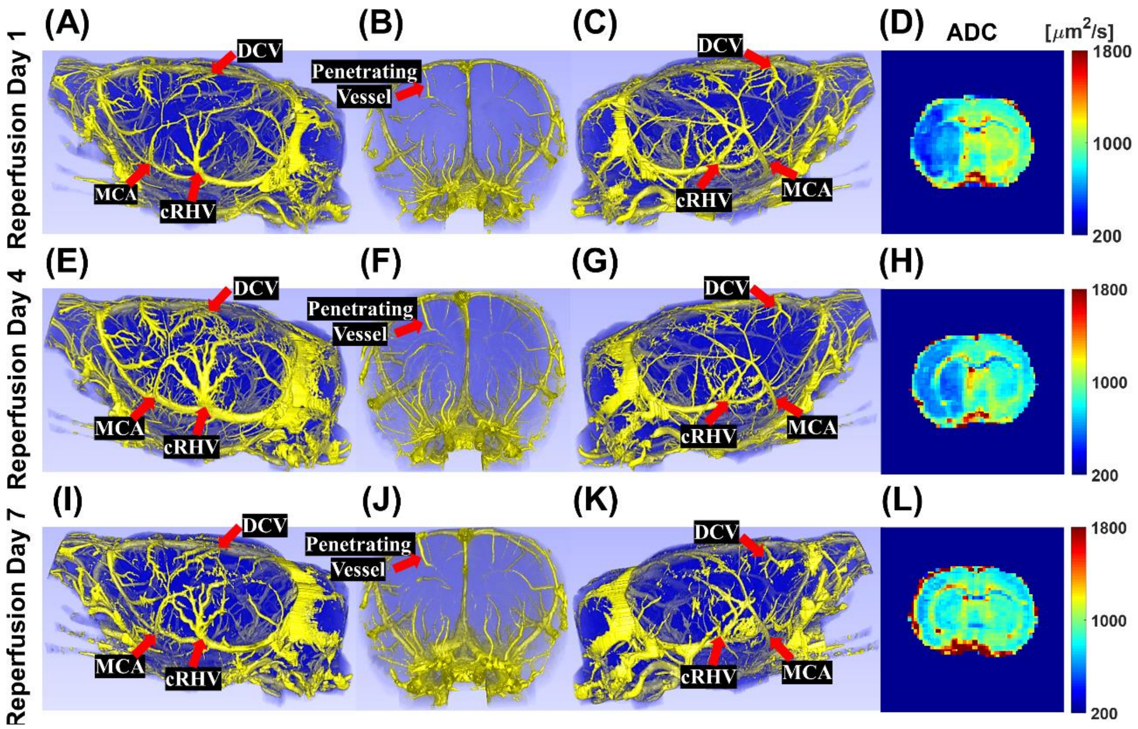

Figure S3. Macrovascular remodeling of tMCAO rat brain. Lateral views of ipsilateral hemisphere (A, E, and I), anterior-to-posterior views (B, F, and J) and lateral views of contralateral hemisphere (C, G, and K) of UTE-MRAs and ADC maps (D, H, and L) acquired at reperfusion days 1, 4, and 7, respectively. Figures (A)-(C), (E)-(G), and (I)-(K) were generated from 3DSlicer (4.5.0-1, www.slicer.org). Figures (D), (H), and (L) were generated from MATLAB (R2017b, www.mathworks.com).

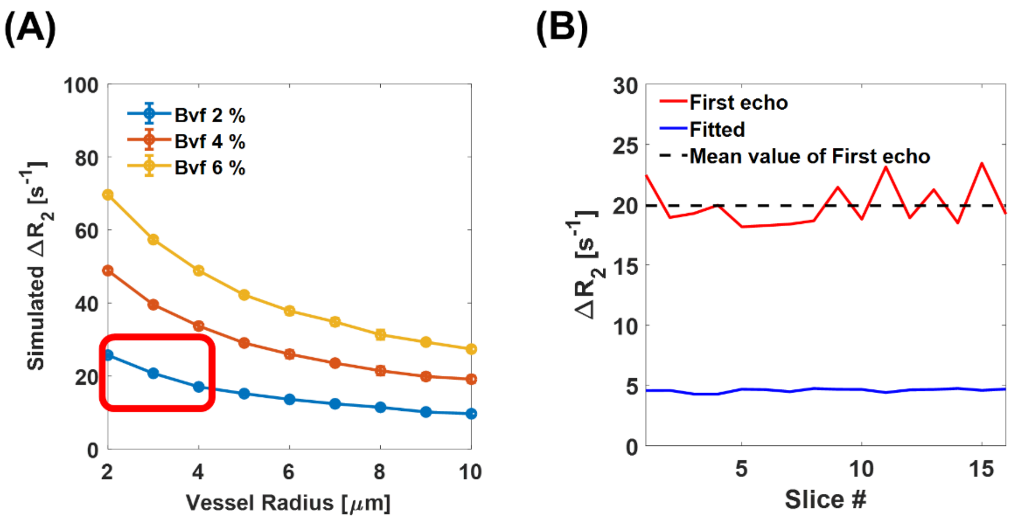

Figure S4. ΔR_2 value calibration. Simulated ΔR_2 values (A, red box) and corresponding values obtained from the first echo of MSME acquisition were consistent (B, red line). The ΔR_2 values obtained from the fitted echo trains of MSME acquisition (B, blue line). The ratio between ΔR_2 values first echo and fitted echo trains was 4.375 and this ratio was calibrated for VSI/Q/MVD calculation.

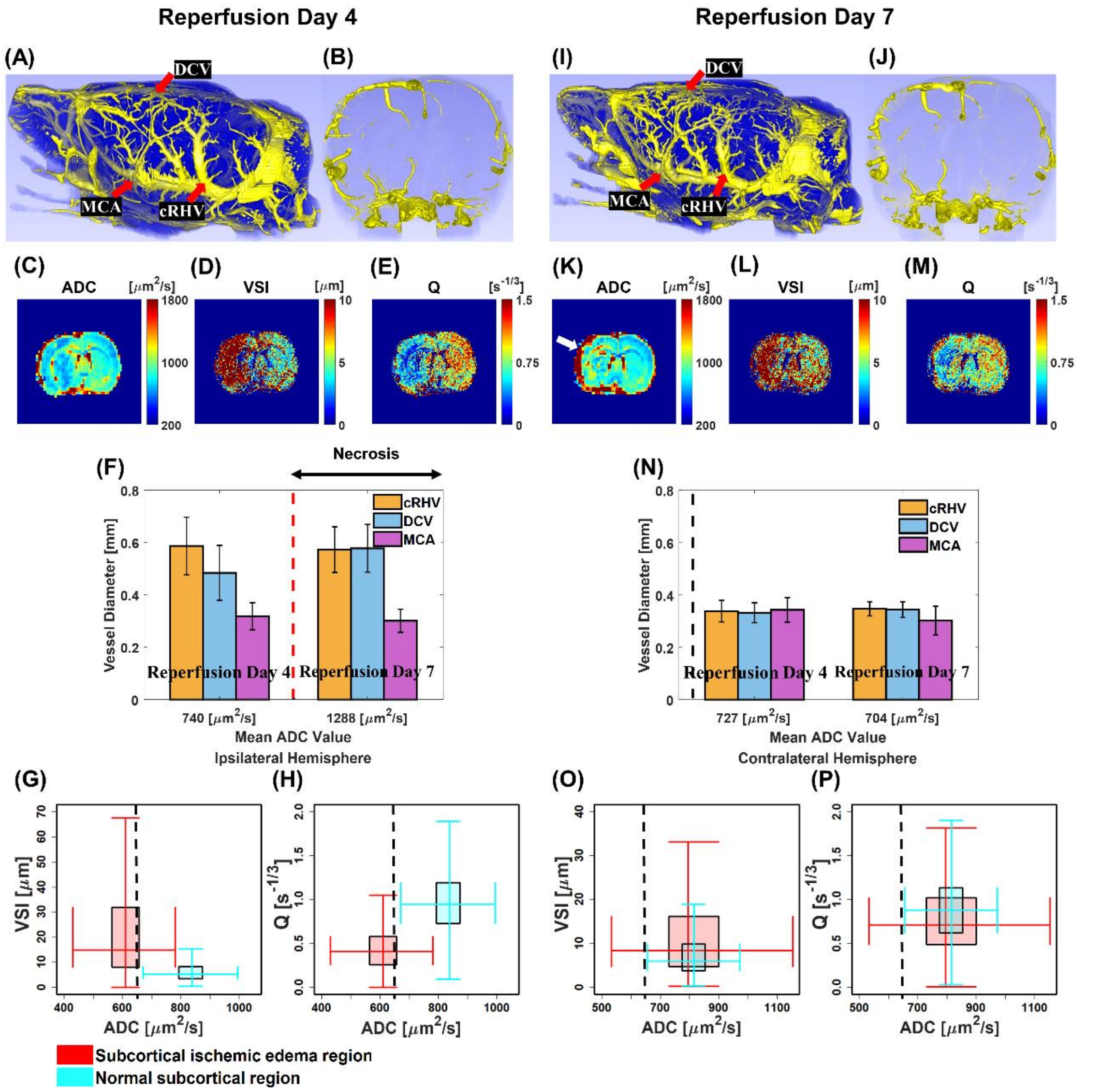

Figure S5. Macro- and microvascular remodeling of tMCAO rat brain. Lateral views of ipsilateral hemisphere (A and I) and anterior-to-posterior views (B and J) of tMCAO rat brain UTE-MRAs and corresponding ADC maps (C and K), VSI maps (D and L), and Q

maps (E and M) of tMCAO rat brain acquired at reperfusion days 4 and 7, respectively. Vessel diameters of cRHV (orange), DCV (blue), and MCA (purple) of the ipsilateral hemisphere with respect to mean ADC values of the corresponding cortical lesion at reperfusion days 4 and 7 (F). Vessel diameters of cRHV (orange), DCV (blue), and MCA (purple) of the contralateral hemisphere with respect to mean ADC values of the corresponding cortical region at reperfusion days 4 and 7 (N). Double box plots between VSI values and corresponding ADC values of subcortex (G and O) for reperfusion days 4 and 7, respectively. Double box plots between Q values and corresponding ADC values of subcortex (H and P) for reperfusion days 4 and 7, respectively. The black dotted vertical lines mark the ADC = 650 $\mu m^2/s$, which are set to separate ischemic edema from recovered/normal tissue. The red dotted vertical line in (F) separates recovered tissue from necrotic tissue. Figures (A), (B), (I), and (J) were generated from 3DSlicer (4.5.0-1, www.slicer.org). Figures (C)-(E) and (K)-(M) were generated from MATLAB (R2017b, www.mathworks.com).

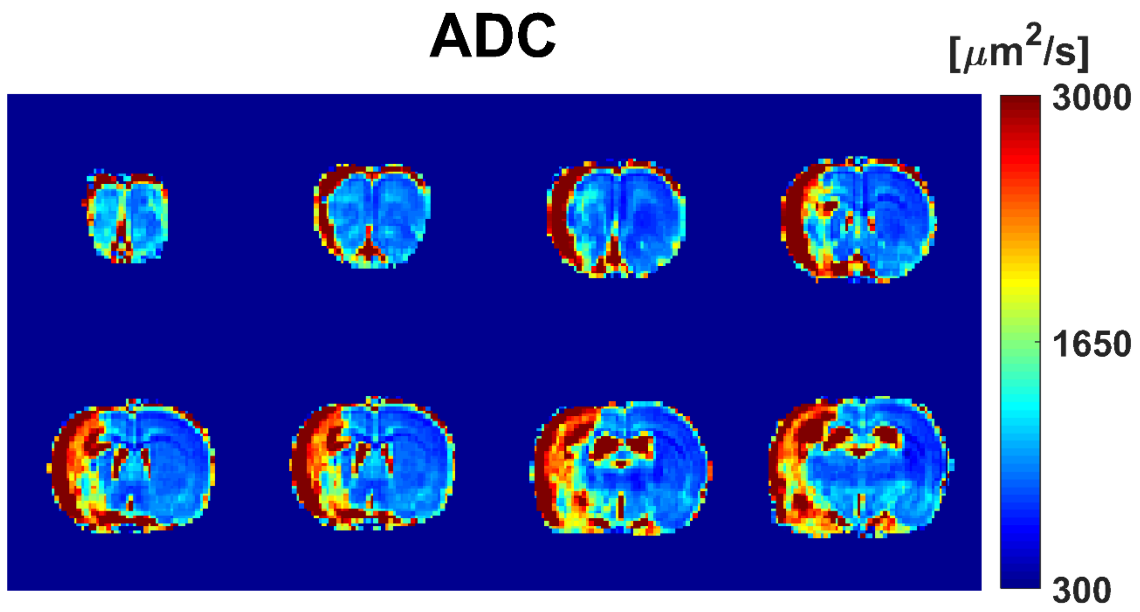

Figure S6. ADC maps of tMCAO rat brain acquired at reperfusion day 13 (same tMCAO rat model as in Supplementary Fig. S5). Figure was generated from MATLAB (R2017b, www.mathworks.com).

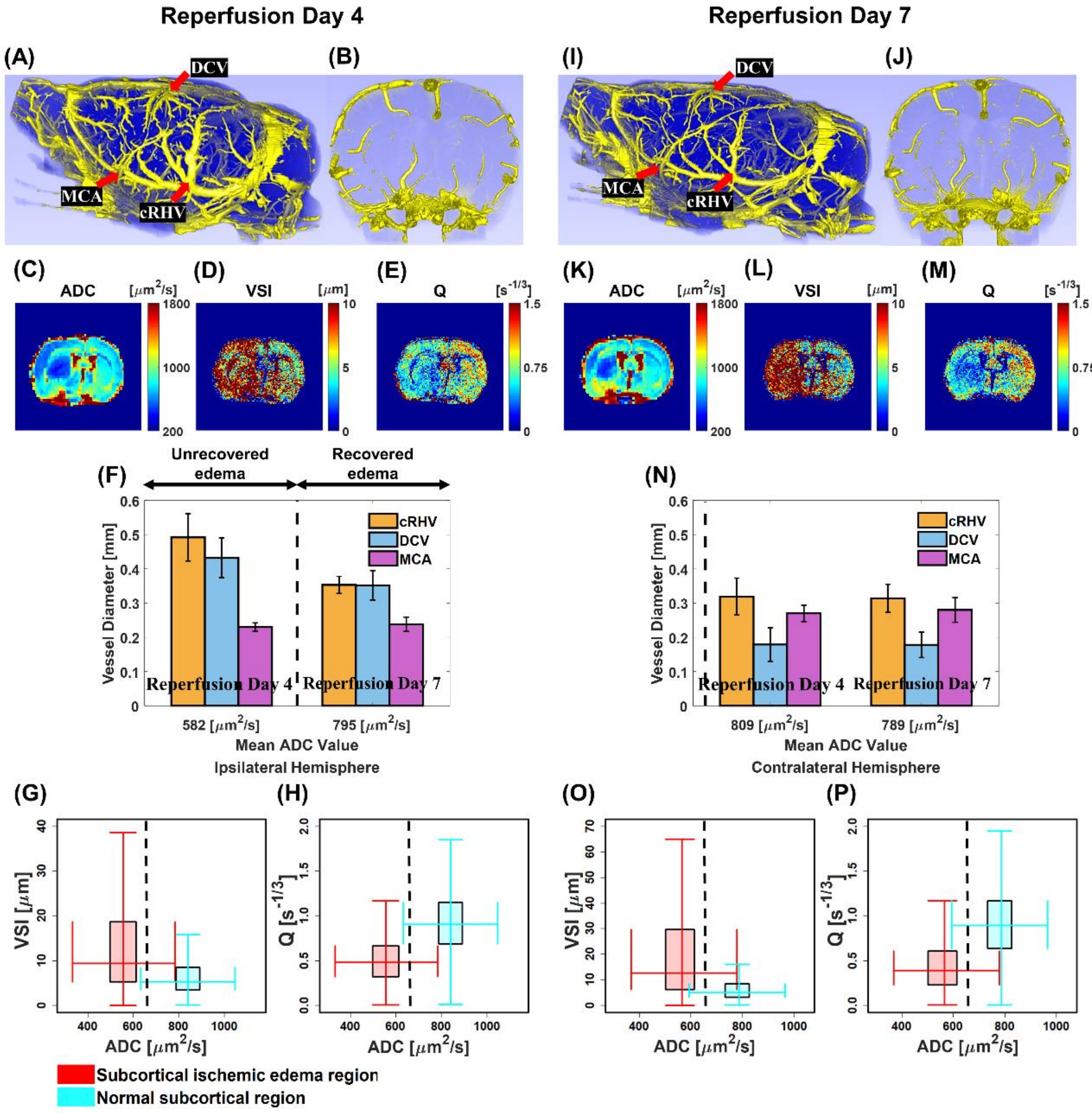

Figure S7. Macro- and microvascular remodeling of tMCAO rat brain. Lateral views of ipsilateral hemisphere (A and I) and anterior-to-posterior views (B and J) of tMCAO rat brain UTE-MRAs and corresponding ADC maps (C and K), VSI maps (D and L), and Q maps (E and M) of tMCAO rat brain acquired at reperfusion days 4 and 7, respectively.

Vessel diameters of cRHV (orange), DCV (blue), and MCA (purple) of the ipsilateral hemisphere with respect to mean ADC values of the corresponding cortical lesion at reperfusion days 4 and 7 (F). Vessel diameters of cRHV (orange), DCV (blue), and MCA (purple) of the contralateral hemisphere with respect to mean ADC values of the corresponding cortical region at reperfusion days 4 and 7 (N). Double box plots between VSI values and corresponding ADC values of subcortex (G and O) for reperfusion days 4 and 7, respectively. Double box plots between Q values and corresponding ADC values of subcortex (H and P) for reperfusion days 4 and 7, respectively. The dotted vertical lines mark the ADC = 650 $\mu m^2/s$, which are set to separate ischemic edema from recovered/normal tissue. Figures (A), (B), (I), and (J) were generated from 3DSlicer (4.5.0-1, www.slicer.org). Figures (C)-(E) and (K)-(M) were generated from MATLAB (R2017b, www.mathworks.com).
